# Addressing the Impact of Climate Change on Sexual and Reproductive Health Among Adolescent Girls and Young Women in Low- and Middle-Income Countries

**DOI:** 10.9745/GHSP-D-23-00374

**Published:** 2024-02-28

**Authors:** Paul A. Burns, Clive Mutunga

**Affiliations:** a Department of Population Health Science, John D. Bower School of Population Health, University of Mississippi Medical Center, Jackson, MS, USA.; b U.S. Agency for International Development, Bureau for Global Health, Office of Population and Reproductive Health, Division of Research, Technology and Utilization, Washington, DC.; c American Association for the Advancement of Science, Washington, DC.; d BUILD (Building Capacity for Integrated Family Planning & Reproductive Health and Population, Environment and Development Action), The African Institute for Development for Policy, Lilongwe, Malawi.

## Abstract

There is an urgent need to better understand the role of climate change on sexual and reproductive health outcomes, particularly among adolescent girls and young women in low- and middle-income countries. Stakeholders at all levels should apply a rights-based, gendered approach to climate action and adaptation.

## Key Findings

- Adolescent girls and young women (AGYW) residing in low- and middle-income countries (LMICs) are particularly vulnerable to climate-related sexual and reproductive health (SRH) challenges, placing them at increased risk of poverty, food insecurity, school dropout, transactional sex, sexual violence, sexually transmitted infections, early marriage, and early childbearing.
- There is an urgent need for increased research and climate financing to ensure adequate investments toward advancing climate adaptation and innovation to address the sexual and reproductive needs of AGYW living in LMICs.
- Applying a rights-based, multilevel, multisectoral, and gendered approach to climate action can strengthen National Adaptation Plans of Action and improve the health and well-being of adolescent girls and young women living in LMICs and resource-constrained settings.
- Leveraging community engagement through the inclusion of diverse stakeholders, such as national governments, multilaterals, policymakers, health care providers, researchers, and nongovernmental organizations and youth, in climate-related SRH research and policy can lead to more meaningful participation, deeper trust, and sustainable climate-related SRH solutions in LMICs.

## INTRODUCTION

Adolescents and youth are particularly vulnerable to the effects of climate change due to the numerous biological, emotional, and sociocultural developmental changes occurring during the transition from childhood to adulthood.^1^ Climate change affects adolescents’ health and well-being in several domains, including physical health (e.g., brain development, lung conditions, and nutrition), mental health, education, and sexual and reproductive health (SRH).^2^ In addition to having direct effects due to extreme weather, climate change has indirect and long-term consequences on adolescents’ psychological functioning, educational and employment opportunities, and a sense of security about their futures. While the effects of climate change negatively impact all adolescents and youth, those residing in low- and middle-income countries (LMICs) are most vulnerable to those impacts due to exposures generated by extreme weather and exacerbated by rapid urbanization, poor infrastructure, unplanned slums and informal settlements, and political and economic insecurity. Adolescent girls and young women (AGYW) bear a double burden due to their gender and age, placing them at increased risk of poverty, food insecurity, school dropout, transactional sex, sexual violence, sexually transmitted infections (STIs), early marriage (leading to nonconsensual or unwanted but expected sexual activity), or early childbearing due to a lack of access to and use of prevention methods (e.g., contraceptive methods).^3^^–^^11^

Globally, there are approximately 880 million AGYW aged 15–24 years.^12^ Despite accounting for 12% of the world’s population, their voices are often left out of policy conversations that impact their SRH. Moreover, there is a dearth of research examining the role of climate change on adolescent SRH, especially AGYW in LMICs. A scoping review exploring the impacts of climate-driven migration on SRH found only 10 studies that met the inclusion criteria, underscoring a general lack of attention to SRH in climate research.^13^ The original United Nations Framework Convention on Climate Change (UNFCCC) that outlined the global priorities to mitigate the impacts of climate did not mention women and girls or SRH.^14^ The word “health” appears only twice in the entire 25-page, 8,496-word document. However, more recent UNFCCC policy documents have highlighted the importance of gender equity in addressing climate change.^15^^–^^17^ It was not until 2002, at the 52nd Session of the Intergovernmental Panel on Climate Change, that the Panel adopted the Gender Policy and Implementation Plan.^17^^,^^18^ Then, in 2018, the Guttmacher-Lancet Commission published a report that called for the adoption of a more comprehensive integrated approach to sexual and reproductive rights as well as the recognition of the importance of addressing climate change in the context of SRH.^19^

Although there has been an increasing recognition linking climate change and SRH, there is limited research, programs, and policies that address this issue at the national level in LMICs. In 2002, the National Adaptation Plan of Action (NAP) was established by the UNFCCC under the Cancun Adaptation Framework^20^ and reemphasized in the Paris Agreement^21^ the need to address vulnerability to climate change by building climate adaptative capacity and the integration of adaptation into new and existing policies and programs.^22^ Under the UNFCCC, NAPs are funded through a mechanism managed by the Global Environment Facility to assist LMICs in their efforts to adapt to climate impacts. NAPs provide: (1) a useful vehicle for countries to set climate change priorities, identify medium- and long-term adaptation goals, and develop implementation strategies for action; (2) a systematic approach toward the development of evidence-based findings to support climate adaptive programming and policies; and (3) an opportunity to coordinate and harmonize national adaptation efforts across sectors by including diverse actors, such as government agencies, the private sector, civil society, and other relevant stakeholders, to achieve climate adaptation goals. However, only a few NAPs focus on health, with even fewer addressing the impacts of climate change on SRH. A recent World Health Organization assessment of NAPs submitted to NAP Central, the UNFCCC NAP portal, found all NAPs highlight health as a high-priority sector vulnerable to climate change, but none addressed SRH.^23^ Additionally, another review examining NAP quality found that many countries are not taking a multisectoral approach, which can negatively impact the overall success of the process and outcomes.^24^

## EXAMINING THE IMPACTS OF CLIMATE CHANGE ON SEXUAL AND REPRODUCTIVE HEALTH

### Extreme Weather Events

Climate change poses detrimental impacts on broader ecosystems, exacerbating pressures on global food security, water resources, and livelihoods. Vulnerable populations who contribute least to the climate change crisis are often more impacted.^25^^–^^27^ According to the 2020 Lancet Countdown report, climate-induced extreme weather events and rising sea levels will increase human migration and displacement, posing severe risks to human health, including SRH.^25^^,^^28^^,^^29^ Climate-induced droughts increase food and water insecurity, disrupting livelihoods and ways of life and displacing millions of vulnerable people, particularly in LMICs.^30^^–^^34^ Extreme weather events and climate change can often lead to short- and long-term migration, which has been associated with poverty and increased engagement in transactional sex and sexual exploitation (e.g., early sexual debut and child marriage).^33^

Several socioecological frameworks have been developed to explain how climate-induced resource scarcity (e.g., water and food insecurity) is linked to SRH outcomes (e.g., HIV and STIs). Leiber et al. developed a model to conceptualize pathways and mechanisms for understanding the impact of climate change on SRH, particularly HIV (Figure).^35^ Extreme weather events, such as drought and infrastructure erosion, can reduce access to SRH services^36^; increase rates of sexual risk behavior, including condomless sex and transactional sex^37^^,^^38^; and lead to early sexual debut,^39^ higher prevalence of infectious diseases (e.g., dengue, chikungunya, and malaria) due to changes in season duration,^40^^–^^42^ and migration-related sexual abuse and exploitation.^39^^,^^43^

Extreme weather events can reduce access to SRH services, increase rates of sexual risk behavior, and lead to early sexual debut, higher prevalence of infectious diseases, and sexual abuse and exploitation.

**FIGURE:**
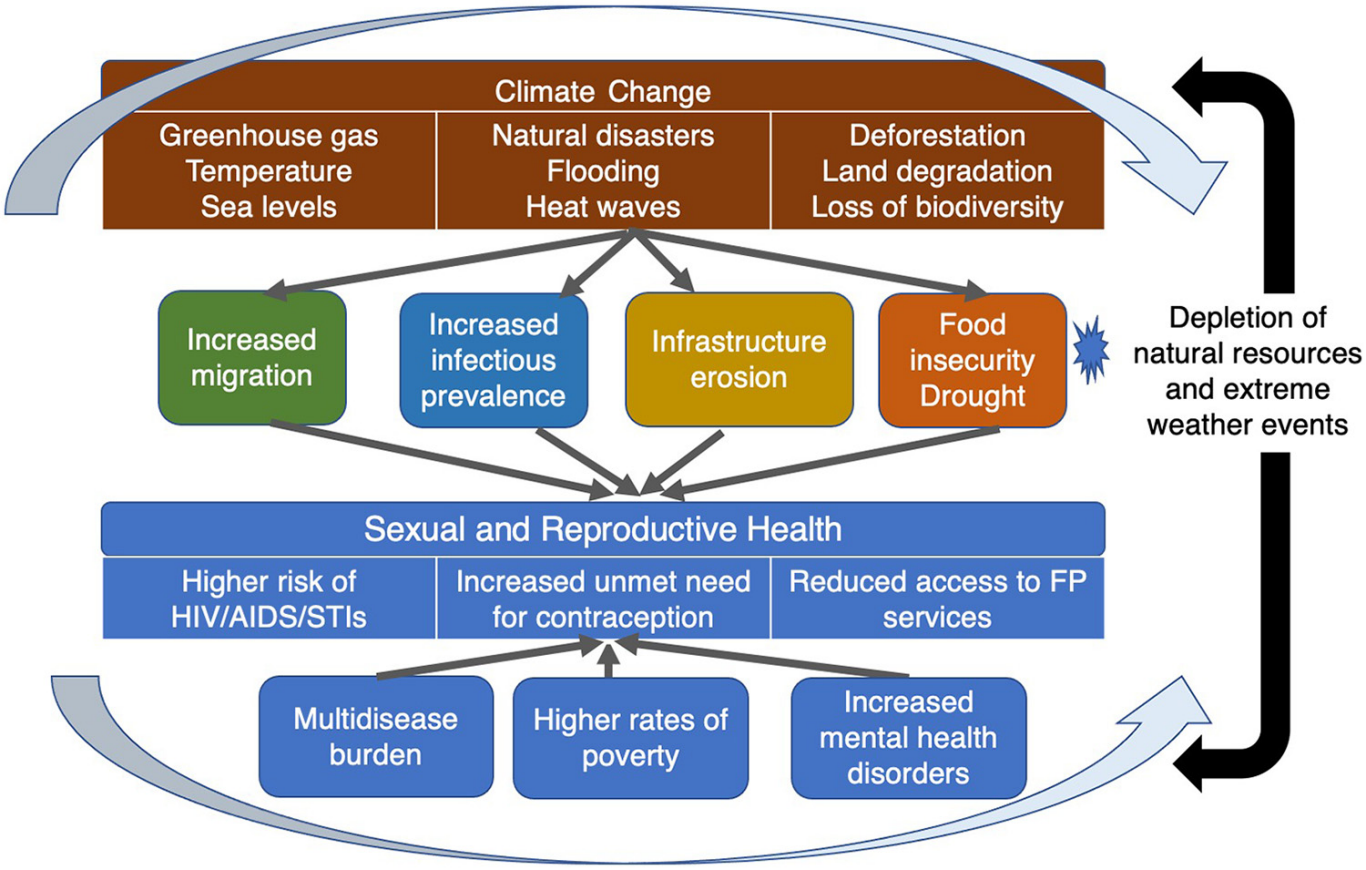
Conceptual Framework for Explaining Links Between Sexual Reproductive Health and Climate Change^a^ Abbreviation: FP, family planning; STI, sexually transmitted infection. ^a^ Adapted from Lieber et al.^35^

The uprooting of communities due to climate-related migration can also disrupt access to and provision of lifesaving SRH services and programs. A growing body of work has linked climate change and extreme weather events to increased risk of HIV and other STIs, particularly for AGYW in LMICs. A study of 21 sub-Saharan African countries comprising nearly 300,000 respondents examining the impacts of long-term temperature increases on HIV found long periods of droughts were associated with increased HIV prevalence. The study also showed droughts were more likely to impact those people living in rural areas, younger age groups, and increased engagement in transactional sex.^43^ In another study of 19 sub-Saharan African countries, Burke et al. found a rainfall shock was associated with an 11% increase in HIV prevalence among rural populations.^39^ Also, a study in Lesotho found adolescent girls who lived in areas that experienced drought in the last 2 years were more likely to have earlier sexual debut and be HIV-positive.^37^ It is also important to note many parents often view their migration-related decisions as protective against the impacts of climate change and not as catalysts for the development of a new set of risks.

### Food Insecurity

Food insecurity has been linked to several adverse health outcomes, including HIV-related clinical outcomes.^44^^–^^46^ Several studies have documented an association between climate-related food insecurity and HIV vulnerability through sexual risk behavior pathways (e.g., survival sex and transactional sex) and mental health (e.g., depression and substance disorders), which can increase exposure to HIV. In sub-Saharan Africa, food insecurity has been linked to sexual risk behavior and transactional sex among HIV-positive youth.^47^^,^^48^ In addition, food insecurity has been shown to reduce condom use among adults and condom efficacy among adolescents^.^^49^^,^^50^ Food insecurity is associated with diminished psychosocial coping resources, leading to greater psychological distress, which may impact a youth’s ability to engage in safer sex practices, including condom use and negotiating condom use with their partners.

### Water Insecurity

Moreover, drought has been linked to HIV vulnerability through its impact on livelihoods through reduced income.^51^^–^^54^ A study in Western Kenya found water insecurity was associated with lower odds of viral suppression and increased odds of acquisition of AIDS-defining illnesses among adults.^55^ Also, lack of access to water may limit an individual’s ability to effectively engage in hygiene and sanitation practices, such as consistent handwashing and bathing.^56^^,^^57^ Evidence suggests water insecurity may also lead to dehydration and fatigue and increase the risk of opportunistic infections from waterborne infections, such as diarrheal or skin diseases.^58^^,^^59^ Exposure to climate-induced AIDS-defining illnesses and infections may, in turn, limit an individual’s ability to maintain adherence to antiretroviral therapy and engagement in HIV care, resulting in lower odds of viral suppression. Water insecurity may also increase stress and worsen mental health outcomes,^60^^–^^65^ leading to lower antiretroviral therapy adherence, engagement in care, and viral load suppression.^47^^,^^57^ In addition, several studies have shown an association between water insecurity and mental health outcomes (e.g., depression and substance use).^58^^,^^62^^,^^63^ Further, the opportunity costs and injuries associated with acquiring water in low-resource settings may limit an individual’s health-seeking behavior as it relates to HIV care.^64^^,^^65^ Climate change also has complex relationships with HIV, particularly for persons living with HIV. Evidence suggests the effects of climate change can compromise the immune systems of people living with HIV, increasing exposure to infectious diseases, disruptions in access to health care, and food insecurity.^47^^,^^66^^–^^72^

### Family Planning and Population Health

One of the key goals of climate adaptation policies is to limit human-derived greenhouse gas emissions. Evidence suggests climate change may exacerbate factors (e.g., socioeconomic mobility, education, employment, and land tenure) associated with uptake and access to family planning services. Population growth exacerbates the threats posed by climate change as a result of natural resource depletion, economic and food insecurity, and poorer health outcomes. However, family planning and SRH within climate adaptive plans are largely ignored, particularly in LMICs.^73^ Through investments in SRH and health literacy programs, we can enhance climate resilience and adaptive capacity at the individual, community, and structural levels. Improving access to effective voluntary modern contraceptive methods can be an important component of a multisectoral approach to climate change adaptation. Although there should be increased investments to address unmet family planning needs in LMICs, these efforts must be in concert with countries in high-income countries (HICs). For example, we must be cognizant of the disproportionate impact of family planning decisions (i.e., unintended pregnancies) in HICs.^74^ Estimates suggest the wealthiest 10% of the global population contributes 50% of greenhouse emissions compared to the poorest 50% contributing only 10% of emissions.^75^ Given the U.S. and UK carbon emissions per capita are more than 150 and 80 times greater, respectively, than most countries in sub-Saharan Africa, any successful climate adaptation policy must be done in concert with climate adaptation plans that address climate change issues in HICs.

## POLICY RECOMMENDATIONS TO MITIGATE THE IMPACT OF CLIMATE CHANGE ON SEXUAL AND REPRODUCTIVE HEALTH

There is an urgent need to increase investments in integrated SRH and climate adaptive approaches to mitigate the impacts of climate change on the SRH of young women and girls. We hope the following 5 recommendations will be useful to governments, multilateral institutions, public health officials, the private sector, civil society, climate advocates, and community leaders in their efforts to design and implement climate adaptive programs and policies, such as the NAPs.

There is an urgent need to increase investments in integrated SRH and climate adaptive approaches to mitigate the impacts of climate change on the SRH of young women and girls.

### 1. Incorporate Sexual and Reproductive Health Into National Adaptive Plans

National governments play a pivotal role in mitigating the effects of climate change. National adaptation planning was designed to provide national governments with an evidence-based, coordinated, and systematic approach to developing and implementing climate preparedness initiatives. However, recent evaluations suggest great variation in their design, content, and implementation. Also, few NAPs include public health initiatives, and none address the SRH of AGYW. We call on national governments to incorporate innovative, multilevel programs and policies that directly address the impacts of climate change on SRH of AGYW living in LMICs.

### 2. Apply a Rights-Based, Gendered Approach to Climate Adaptation

Rights-based climate-related programs and policies centering the lives and SRH needs of AGYW are needed to promote women’s empowerment, economic growth, resilience, and capacity-building. Research has shown extreme weather events due to climate change disproportionately affect women and girls in LMICs, impacting their ability to perform everyday tasks, such as collecting water, cooking, and planting food. Food and water insecurity and loss of livelihoods create the conditions where AGYW are forced to drop out of school, to engage in transactional sex, or to enter into child marriages, which may increase rates of gender-based violence and exposure to HIV and STIs.

NAPs must take a rights-based, gender-responsive approach to climate change by creating inclusive policies in education, employment, laws, land tenure, and health, including SRH and rights, particularly the right to make informed decisions about their sexuality and reproduction, access to SRH services, equal access to educational and career opportunities, and decision-making about their lives and future. More inclusive, rights-based, gender-responsive climate policies promoted by international institutions and national governments can lead to more effective and sustainable climate solutions that reduce environmental harm. The Box lists climate adaptive policies and resources for advancing policy dialogue and policy action around gender and SRH for AGYW in LMICs.^14^^,^^17^^,^^76^^,^^77^

BOX Gender, Sexual and Reproductive Health, and Climate Change Resources

- The United Nations Framework Convention on Climate Change^14^ is the parent treaty of the 2015 Paris Agreement.^21^
- The Gender Action Plan Plan^17^ provides objectives and activities to advance knowledge and understanding of gender-responsive climate action and its coherent mainstreaming in climate adaptive plans and policies.
- Climate Change 2023: Synthesis Report^76^ provides an overview of the state of knowledge concerning the science of climate change referencing sexual and reproductive health.
- Making the Connection: Population Dynamics and Climate Compatible Development^77^ provides approaches and opportunities for advancing policy dialogue and policy action to include population dynamics, with an emphasis on family planning, into climate-compatible development.

### 3. Build Capacity for Integrated Climate Change and Sexual and Reproductive Health

Mitigating climate risks will require multilevel, multisector coordination, improvements in data quality and collection methods, monitoring and evaluation of SRH targets, and inclusive and diverse stakeholder engagement, especially women and girls. In addition, empowering women as agents of social change can improve mitigation and adaptation policies and the design and implementation of gender-responsive climate-related interventions and programs that address impacts on their SRH. The Table provides some examples of initiatives that integrate gender, SRH, and climate change approaches. For example, in Southern Africa, Building Capacity for Integrated Family Planning & Reproductive Health and Population, Environment and Development Action uses a gender-responsive approach to climate adaptation. The project aims to strengthen the capacity of individuals and institutions through population, health, and environment approaches and to boost political and financial commitment to family planning and integrated population health and environment approaches that increase gender equality and youth empowerment.

**TABLE.** Selected Integrated Sexual and Reproductive Health and Climate Change Initiatives

**Name**	**Approach**	**Country/Region**	**Agency**	**More Info**
BUILD: Building Capacity for Integrated Family Planning & Reproductive Health and Population, Environment and Development Action	Increasing commitment to voluntary family planning and reproductive health for climate adaptation	Southern and East Africa, Philippines	USAID, PATH Foundation Philippines, Inc.	https://populationconnection.org/resources/population-health-environment-philippines
Health of People and Environment in the Lake Victoria Basin	Integration of family planning, population, and climate change	Kenya, Uganda	Margaret A. Cargill Foundation, The David and Lucile Packard Foundation, Barr Foundation, John D. and Catherine T. MacArthur Foundation, USAID through the Evidence to Action project	https://www.pathfinder.org/projects/hope-lvb
Population, Environment, and Development	Integrated approach to sexual and reproductive health and climate change	Global initiative	USAID	https://www.usaid.gov/global-health/health-areas/family-planning/resources/helping-people-planet-flourish-family-planning
Drawdown Lift	Integrated approach to girls’ education and family planning	Sub-Saharan Africa and Asia	Corporate sponsors and foundations	https://drawdown.org/publications/drawdown-lift-policy-brief-girls-education-and-family-planning

Abbreviation: USAID, U.S. Agency for International Development.

### 4. Leverage Community Engagement for Gender-Responsive Climate Change Adaptive Policies

Community engagement is vital for the planning and implementation of effective NAPs. To be successful, governments need to create an enabling environment that supports climate adaptation by making commitments to engage a diverse set of stakeholders (e.g., government ministries, elected officials, health care workers, and community members, particularly AGYW) to generate public engagement and attention toward the development of gender-responsive policies to mitigate the impacts of climate change. For example, in the Philippines, PATH implemented a program called Empowering Rural Youth with Population Health Environment and Enterprise Development Know-How that engaged a diverse group of stakeholders (e.g., national government, civil society, community leaders, and local youth) to implement integrated family planning and climate change initiatives, including voluntary family planning campaigns, coastal conservation, and economic development activities that would reduce dependency on fishing. These engagements must be inclusive, values-oriented, and framed to incorporate indigenous knowledge, capacities, and the lived experiences of all citizens, particularly those most impacted by climate change in LMICs.

### 5. Accelerate Financing for Integrated Family Planning and Climate Adaptive Action

Donor countries contribute the bulk of the climate financing through a trust fund administered by the World Bank, serving as the Global Environment Facility Trustee. As of September 30, 2022, 28 donors pledged and signed Contribution Agreements amounting to more than US$1,971.46 million. A recent analysis conducted by UNFCCC found that the financing needs of nationally determined contributions of 78 countries amounted to around US$5.8 trillion until 2030 (or about US$600 billion per year). We call on multilateral organizations, HICs, and the private sector to increase funding to ensure adequate funding for advancing innovation and research—including private-sector financing to ensure effective environmental preparedness and long-term sustainable climate adaptive policies—that addresses the needs of vulnerable countries and populations, particularly, the SRH needs of AGYW residing in LMICs.

## CONCLUSIONS

Despite growing recognition of the relationship between climate change and health, there is limited research examining the links between climate change and SRH among AGYW in LMICs. Additionally, few NAPs address the climate-related SRH needs of AGYW in LMICs. Using the evidence-based findings from the literature and models presented in this article, we call on national governments, international institutions, policymakers, civil society, and private sector actors to: (1) apply a multilevel gendered approach to climate action; (2) employ a rights-based, gendered approach to climate adaptation; (3) build capacity for integrated climate change and SRH; (4) leverage community engagement for gender-responsive climate change adaptive policies; and (5) accelerate financing for integrated family planning and climate adaptive action.

## Notes

**Peer Reviewed**

**First published online:** February 17, 2024.
